# Comparison of Machine Learning Methods With National Cardiovascular Data Registry Models for Prediction of Risk of Bleeding After Percutaneous Coronary Intervention

**DOI:** 10.1001/jamanetworkopen.2019.6835

**Published:** 2019-07-10

**Authors:** Bobak J. Mortazavi, Emily M. Bucholz, Nihar R. Desai, Chenxi Huang, Jeptha P. Curtis, Frederick A. Masoudi, Richard E. Shaw, Sahand N. Negahban, Harlan M. Krumholz

**Affiliations:** 1 Department of Computer Science and Engineering, Texas A&M University, College Station; 2 Center for Remote Health Technologies and Systems, Texas A&M University, College Station; 3 Section of Cardiovascular Medicine, Department of Internal Medicine, Yale School of Medicine, New Haven, Connecticut; 4 Center for Outcomes Research and Evaluation, Yale New Haven Hospital, New Haven, Connecticut; 5 Division of Cardiovascular Medicine, Department of Internal Medicine, Yale School of Medicine, New Haven, Connecticut; 6 Now with the Department of Pediatrics, Boston Children’s Hospital, Boston, Massachusetts; 7 Division of Cardiology, Department of Medicine, University of Colorado Anschutz Medical Campus, Aurora; 8 Division of Cardiology, Department of Medicine, California Pacific Medical Center, Sutter Health, San Francisco; 9 Department of Statistics, Yale University, New Haven, Connecticut; 10 Department of Health Policy and Management, Yale School of Public Health, New Haven, Connecticut

## Abstract

**Importance:**

Better prediction of major bleeding after percutaneous coronary intervention (PCI) may improve clinical decisions aimed to reduce bleeding risk. Machine learning techniques, bolstered by better selection of variables, hold promise for enhancing prediction.

**Objective:**

To determine whether machine learning techniques better predict post-PCI major bleeding compared with the existing National Cardiovascular Data Registry (NCDR) models.

**Design, Setting, and Participants:**

This comparative effectiveness study used the NCDR CathPCI Registry data version 4.4 (July 1, 2009, to April 1, 2015), machine learning techniques were used (logistic regression with lasso regularization and gradient descent boosting [XGBoost, version 0.71.2]), and output was then compared with the existing simplified risk score and full NCDR models. The existing models were recreated, and then performance was evaluated through additional techniques and variables in a 5-fold cross-validation in analysis conducted from October 1, 2015, to October 27, 2017. The setting was retrospective modeling of a nationwide clinical registry of PCI. Participants were all patients undergoing PCI. Percutaneous coronary intervention procedures were excluded if they were not the index PCI of admission, if the hospital site had missing outcomes measures, or if the patient underwent subsequent coronary artery bypass grafting.

**Exposures:**

Clinical variables available at admission and diagnostic coronary angiography data were used to determine the severity and complexity of presentation.

**Main Outcomes and Measures:**

The main outcome was in-hospital major bleeding within 72 hours after PCI. Results were evaluated by comparing C statistics, calibration, and decision threshold–based metrics, including the *F* score (harmonic mean of positive predictive value and sensitivity) and the false discovery rate.

**Results:**

The post-PCI major bleeding rate among 3 316 465 procedures (patients’ median age, 65 years; interquartile range, 56-73 years; 68.1% male) was 4.5%. The existing full model achieved a mean C statistic of 0.78 (95% CI, 0.78-0.78). The use of XGBoost and full range of selected variables achieved a C statistic of 0.82 (95% CI, 0.82-0.82), with an *F* score of 0.31 (95% CI, 0.30-0.31). XGBoost correctly identified an additional 3.7% of cases identified as high risk who experienced a bleeding event and an overall improvement of 1.0% of cases identified as low risk who did not experience a bleeding event. The data-driven decision threshold helped improve the false discovery rate of the existing techniques. The existing simplified risk score model improved the false discovery rate from more than 90% to 78.7%. Modifying the model and the data decision threshold improved this rate from 78.7% to 73.4%.

**Conclusions and Relevance:**

Machine learning techniques improved the prediction of major bleeding after PCI. These techniques may help to better identify patients who would benefit most from strategies to reduce bleeding risk.

## Key Points

**Question:**

Can machine learning techniques, bolstered by better selection of variables, improve prediction of major bleeding after percutaneous coronary intervention (PCI)?

**Findings:**

In this comparative effectiveness study that modeled more than 3 million PCI procedures, machine learning techniques improved the prediction of post-PCI major bleeding to a C statistic of 0.82 compared with a C statistic of 0.78 from the existing model. Machine learning techniques improved the identification of an additional 3.7% of bleeding cases and 1.0% of nonbleeding cases.

**Meaning:**

By leveraging more complex, raw variables, machine learning techniques are better able to identify patients at risk for major bleeding and who can benefit from bleeding avoidance therapies.

## Teaser

This comparative effectiveness study of a nationwide clinical registry of percutaneous coronary intervention (PCI) determines whether machine learning techniques better predict post-PCI major bleeding compared with the existing National Cardiovascular Data Registry (NCDR) models.

## Introduction

Major bleeding, a common complication after percutaneous coronary intervention (PCI), is associated with increased mortality risk, other periprocedural complications, and greater cost.^1,2,3,4,5^ Several risk prediction models and bleeding prevention strategies have been developed to identify patients who are at highest risk for post-PCI bleeding and may benefit from bleeding avoidance therapies. In particular, 2 models from the National Cardiovascular Data Registry (NCDR) have been widely used for risk stratification and quality improvement initiatives.^1,3^ Rao et al^3^ developed and then updated the following 2 NCDR bleeding risk models: (1) a 31-variable existing full PCI model that uses 23 patient characteristic variables (available at presentation) and 8 procedural characteristics focused on the coronary anatomy and culprit lesion to aid in clinical decisions about bleeding avoidance strategies, and (2) an existing simplified risk score model using 10 pre-PCI variables selected from the 31 variables chosen for the existing full model. Many of the 31 variables were presented as dichotomous variables extracted from continuous variables. The existing full model was derived using logistic regression with backward elimination of the available NCDR CathPCI Registry data.^3^

Although these NCDR bleeding risk models have performed well in validation cohorts and in clinical practice, the current model discrimination of 0.77 leaves room for an improvement in the definition of high-risk patients. While decision thresholds can be varied to determine treatment decision paradigms, the existing models use low thresholds for high risk, resulting in classifying an abundant number of nonbleeding cases as high risk. Therefore, we examined the improvement in model discrimination.

Machine learning techniques may enhance risk prediction because they allow nonlinear associations and are better suited to extracting additional information from continuous variables (eg, preprocedural hemoglobin continuous value rather than 2 variables of preprocedural hemoglobin [≤13 and >13 g/dL]) (to convert hemoglobin level to grams per liter, multiply by 10.0). Prior work shows that machine learning techniques can boost clinical prediction if the appropriate data are available.^6^ If machine learning techniques substantially improve risk prediction, they may be able to improve clinical decision making before and during PCI. The American College of Cardiology uses the existing model,^3^ indicating support for improvements of such a model that can be used for addressing clinical decision making and be used for quality improvement by improving risk adjustment.

This work investigates a direct comparison of modeling techniques with the existing full PCI model and the existing simplified risk score. We conducted 4 data experiments to determine if machine learning algorithms can meaningfully improve risk prediction and whether the preparing of the data as dichotomous variables altered the ability to improve the models. These experiments included an analysis of (1) the association machine learning techniques have with the existing simplified risk score, (2) the association the machine learning techniques have with the existing full model with the same selected variables, (3) the association the machine learning techniques have with the existing full model when we allow the model to examine the full dynamic range of the variables (blended model), and (4) our understanding of the importance of variables in the best-performing (blended) model. We purposely constrained this study to variables in the current model and the underlying data that were used to produce those variables.

## Methods

### Study Population

The analysis used data from version 4.4 of the NCDR CathPCI Registry,^3,7^ which includes PCIs performed from July 1, 2009, to April 1, 2015. This registry, cosponsored by the American College of Cardiology and the Society for Cardiovascular Angiography and Interventions, includes data on patient characteristics, clinical features, angiographic and procedural details, and in-hospital outcomes on interventional cases at 1561 participating institutions. Data quality is monitored through extensive data abstraction training, site feedback reports, independent auditing, and data validation, reducing the likelihood of large outliers or high rates of missing data.^4,8^ We conducted this analysis from October 1, 2015, to October 27, 2017, on a deidentified extract in an institutional review board–approved study (Yale Human Research Protection Program); requirement for informed consent was waived owing to the use of deidentified data. The setting was retrospective modeling of a nationwide clinical registry of PCI. Participants were all patients undergoing PCI. Percutaneous coronary intervention procedures were excluded if they were not the index PCI of admission, if the hospital site had missing outcomes measures, or if the patient underwent subsequent coronary artery bypass grafting. This work follows the Transparent Reporting of a Multivariable Prediction Model for Individual Prognosis or Diagnosis (TRIPOD) reporting guidelines.

Our initial sample used inclusion and exclusion criteria for the existing NCDR bleeding risk model (eFigure 1 in the Supplement).^3^ This study population excluded patient admissions that represented readmissions, patients who died in the hospital the same day as the procedure (even if they had a bleeding event before death, as was constrained in the prior work as well), and patients who had missing bleeding information.^3^ We only included the first PCI procedure within the same episode because we have unique coded identifiers per admission and procedure identifiers linked to this. If a patient had a second PCI in a different admission, we treated this as an independent procedure because we did not have patient identifiers. We added an exclusion for patients who underwent coronary artery bypass grafting (CABG) because the high risk of bleeding after CABG may obscure the bleeding risk attributable to PCI alone; these cases were not excluded in the primary prior model.^5^ Rao et al^3^ also evaluated a cohort that excluded patients who underwent CABG, increasing discrimination to 0.78 (from 0.77) for the existing full model and to 0.76 (from 0.75) for the existing simplified risk score.

### Variable Set Creation

We recreated the existing full NCDR bleeding risk model variable set (31 variables updated with the additional participant data samples) and 10-variable existing simplified risk score variable set. In addition, we created 2 extensions to evaluate changes in performance with each variable. First, the blended model variable set includes the 31 variables from the full NCDR bleeding risk model, plus 28 additional variables that were used to derive the dichotomous variables used in the existing full model. While this would create colinear variables, the modeling techniques used are able to select variables while building models, countering this occurrence. Second is the top features variable set, in which the top-ranked variables in the best-performing model are incrementally added to measure the discrimination of the variables.

### Derivation and Validation

We created derivation and validation cohorts using stratified 5-fold cross-validation. Each variable set was divided randomly into 5 equal subsets, preserving the same event rate in each subset, by first randomly dividing bleeding cases and then nonbleeding cases. Each bleeding subset was then paired with 1 nonbleeding subset. The derivation cohort combined 4 (80%) of the subsets; the remaining subset (20%) was reserved as a validation set. This process was repeated 5 times, such that each of the subsets served as the validation set. While results are provided for each patient, models are fairly compared as being trained on 80% of the data and tested on the unseen 20% 5 separate times, and each case has a single risk estimate produced.

### Variable Selection and Imputation

All 59 variables considered in the blended model data set were collected from the NCDR CathPCI Registry. For binary variables, missing values were redefined as “no” values (eg, history of hypertension was considered “yes” only if it was explicitly recorded). Categorical variables, such as the New York Heart Association class I through IV, which lacked a “no” category, were coded as 1, 2, 3, and 4; an additional category of 0 was added, indicating patients for whom a value was not recorded. Therefore, category 0 indicates no heart failure, and categories 1 through 4 correspond to the New York Heart Association classification. For continuous variables, missing values were imputed via a single median imputation, as was done in the existing full model.^3^ All variables had less than 50% missing, with preprocedural left ventricular ejection fraction having the highest missing rate (29.9%), followed by preprocedural hemoglobin (6.6%), glomerular filtration rate (5.6%), preprocedural creatinine (5.6%), and all other variables having little or no missing data (<1%).^3^

### Outcome Definition

Consistent with the existing NCDR CathPCI Registry bleeding risk model, post-PCI major bleeding was defined as any post-PCI, predischarge major bleeding within 72 hours (eAppendix in the Supplement).^3^ The NCDR data and outcomes, audited and derived from medical records, increase reliability by allowing only hospitals that pass quality checks.^8^

### Model Development

Two methods were used to train models in this analysis. First, logistic regression with lasso regularization is a statistical technique selected to show the value of changing automated variable and model selection techniques.^9^ Second, gradient descent boosting was used to demonstrate the power of machine learning techniques that account for higher-order, nonlinear interactions, particularly on a variety of data types, including binary and continuous variables, which selects variables while training. Gradient descent boosting creates a series of “boosted” decision trees of weaker individual predictors to create stronger final predictions, permitting analysis of higher-order interactions with varying variables and types.^9^ The particular method of gradient descent boosting was extreme gradient boosting (XGBoost, version 0.71.2; xgboost developers).^10^ The final model used 1000 trees, a learning rate of 0.1, and a maximum depth of each tree of 6, and it was trained with an objective functioned aimed at minimizing errors similar to logistic regression for binary classification (bleed vs nonbleed). These models, hyperparameters, and how they were selected are further described in the eAppendix in the Supplement.

### Model Evaluation

Receiver operating characteristic curves were used to estimate model discrimination by the C statistic, and the 5-fold cross-validation provides 5 C statistics that allow for a mean C statistic and 95% CI to be calculated. However, because C statistics do not indicate the ideal decision threshold or give patient-specific prediction, we evaluated each model’s patient-specific predictive ability by comparing the correctly and incorrectly identified bleeding cases and nonbleeding cases at a data-driven decision threshold. From these predictions, we calculated the number of true-positive (TP), true-negative (TN), false-positive (FP), and false-negative (FN) results. The positive predictive rate (TP / [TP + FP]) and sensitivity (TP / [TP + FN]) were combined to yield the *F* score (harmonic mean of positive predictive value and sensitivity). The optimal threshold for each model was defined as that which maximized the *F* score along the receiver operating characteristic curve.^11^

We first assessed the associations of machine learning techniques on the existing variable sets. We then extended the experiments to the blended model variable set (and top features set) to evaluate the gains from the variables, as well as methods that can leverage these variables. Using the best-performing model, we then evaluated the model calibration, analyzed the importance of variables, and assessed prediction performance at the data-driven decision thresholds. We used calibration plots to plot the observed bleeding rate for each decile of predicted risk, compared by the Brier score, and with continuous general additive models.^12^

In supplementary analyses (eAppendix in the Supplement), we sought to better understand the updated samples available with the longer date range. Specifically, because our date range does not match that of the prior work, we split analyses by year, for cases considered in the existing models vs newly collected cases, to confirm that changes in bleeding rates do not alter model discrimination (they did not). In addition, we performed supplementary analyses to ensure that our top features selection technique was a fair selector of variables, including avoiding overfitting and data leakage, and includes a discussion of why forward stepwise selection was a fair choice for comparison.

### Software Implementation

All analyses were conducted in R (version 3.3.2; R Project for Statistical Computing), with GLMNET used for lasso regularization,^13^ XGBoost for gradient descent boosting^10^ and pROC for C statistics.^14^ We used mgcv and sandwich for the continuous calibration curves, and SpecsVerification was used for the Brier score.^15,16,17^ Source code is available online.^18^

### Assessment of Predictor Variables

We evaluated the importance of each variable in the best-trained model (XGBoost) using a variety of metrics.^10^ Each training iteration might present a different ordering of variables; therefore, the importance of variables was calculated from a model trained on all the data. We took these selected variables, listed in order of importance, and added each variable in the 5-fold cross-validation data in a forward stepwise selection to identify the mean incremental C statistic. In high-dimensional problems, backward selection techniques may lend themselves to solutions altered by greater noise.^19^ Instead, we focused on forward selection techniques because they have strong theoretical guarantees^19,20^ and excellent empirical behavior.^9^ The eAppendix in the Supplement includes details on ensuring a fair evaluation. Similar C statistics assure that we did not overfit in this additional analysis.

## Results

### Patient Characteristics

This study included 3 316 465 PCI procedures (patients’ median age, 65 years; interquartile range, 56-73 years; 68.1% male) performed at 1538 sites (eFigure 1 in the Supplement). Major bleeding occurred in 4.5% of patients after PCI. Baseline patient characteristics by bleeding status are listed in Table 1. Candidate variables, their definitions and data types, and sources are listed in Table 2.

**Table 1.** Patient Characteristics^a^

Characteristic	Full Sample (N = 3 316 465)	Bleeding Cases (n = 149 724)	Nonbleeding Cases (n = 3 166 741)
Post-PCI major bleeding rate, %	4.5	100	0
Demographic characteristics			
Age, median (IQR), y	65 (56-73)	68 (59-77)	65 (56-73)
Male sex, %	68.1	50.6	68.9
BMI, median (IQR)	29.1 (25.7-33.3)	27.7 (24.3-32.1)	29.2 (25.8-33.4)
Medical conditions			
Diabetes, %	37.0	37.5	36.9
Hypertension, %	82.1	80.2	82.2
Peripheral vascular disease, %	12.2	15.2	12.0
Chronic kidney disease, %	3.7	9.9	3.5
Previous PCI, %	41.2	30.0	41.7
Previous CABG, %	18.1	14.6	18.3
Preprocedural hemoglobin, median (IQR), g/dL	13.7 (12.5-14.8)	13.6 (11.6-14.9)	13.7 (12.5-14.8)
Procedural status, %			
Elective	41.5	19.1	42.6
Urgent	39.9	33.2	40.3
Emergent	18.3	45.9	17.0
Salvage	0.3	1.8	0.2
STEMI	16.7	42.5	15.5
Shock	2.5	14.3	1.9
Cardiac arrest within 24 h of PCI	1.9	10.0	1.5

Abbreviations: BMI, body mass index (calculated as weight in kilograms divided by height in meters squared); CABG, coronary artery bypass grafting; IQR, interquartile range; PCI, percutaneous coronary intervention; STEMI, ST-segment elevation myocardial infarction.

SI conversation factor: To convert hemoglobin level to grams per liter, multiply by 10.0.

^a^ Bleeding cases are the subset of the full sample deemed to have had major bleeding as an outcome in the 72-hour period after PCI. The nonbleeding cases are those who did not have this adverse outcome.

**Table 2.** Names of Variables, Descriptions, and Use in Prior Models

Variable	Description [NCDR CathPCI Registry Field No.]	Use in Prior Model
**Demographic Characteristics and Medical History**		
Age	Patient age [2050]	Existing simplified risk score
Age >70 y	Is patient age >70 y? (yes or no) [2050]	Existing full model
Age ≤70 y	Is patient age ≤70 y? (yes or no) [2050]	Existing full model
BMI	BMI [4055, 4060]	Existing simplified risk score
BMI ≤30	Is BMI ≤30? (yes or no) [4055, 4060]	Existing full model
Chronic lung disease	Chronic lung disease (yes or no) [4080]	Existing full model
Chronic kidney disease	Chronic kidney disease, composite categorical: 1, 2, 3, 4 [4065]	Existing simplified risk score
No chronic kidney disease: 1	Patients with GFR ≥60 mL/min/1.73 m^2^ (yes or no)	NA
Mild chronic kidney disease: 2	Patients with GFR ≥45 and <60 mL/min/1.73 m^2^ (yes or no)	Existing full model
Moderate chronic kidney disease: 3	Patients with GFR ≥30 and <45 mL/min/1.73 m^2^ (yes or no)	Existing full model
Severe chronic kidney disease: 4	Patients with GFR <30 mL/min/1.73 m^2^ or receiving current dialysis (yes or no) [4065]	Existing full model
GFR	GFR (continuous) [7315, 2050, 2060, 2071]	NA
Female sex	Is patient female? (yes or no) [2060]	Existing simplified risk score, existing full model
Diabetes	Diabetes (yes or no) [4085]	NA
Diabetes composite	Diabetes and therapy composite: 1, 2 [4090]	NA
Diabetes with noninsulin treatment: 1	Diabetes therapy (yes or no) [4090]	NA
Insulin-requiring diabetes: 2	Diabetes therapy (yes or no) [4090]	Existing full model
Currently receiving dialysis	Dialysis (yes or no) [4065]	NA
NYHA composite	0 (No), 1 (class I), 2 (class II), 3 (class III), 4 (class IV) [5025, 5045]	NA
NYHA 1, 2, 3	Class IV vs <IV (yes or no) [5045]	Existing full model
NYHA 4	Class IV (yes or no) [5045]	Existing full model
History of cerebrovascular disease	Cerebrovascular disease (yes or no) [4070]	Existing full model
History of peripheral arterial disease	Peripheral arterial disease (yes or no) [4075]	Existing full model
Previous PCI	Previous PCI in prior visit (yes or no) [4035]	Existing simplified risk score, existing full model
**Procedural Characteristics**		
Preprocedural TIMI flow grade	Preprocedural TIMI flow grade: 0, 1, 2, or 3 [7140]	NA
Preprocedural TIMI flow grade: 0	Preprocedural TIMI flow grade is 0 (yes or no) [7140]	Existing full model
PCI lesion composite	1: Proximal right, mid-LAD, or proximal circumflex	NA
	2: Proximal LAD	
	3: Left main	
	0: Other [7105]	
Proximal LAD PCI	PCI is for proximal LAD lesion (yes or no) [7105]	Existing full model
Left main PCI	PCI is for left main lesion (yes or no) [7105]	Existing full model
Vessel disease composite	Vessel disease: 0, 1, 2, or 3 [6100, 6110, 6120, 6130, 6140, 6150, 6160]	NA
2-Vessel or 3-vessel disease	2-Vessel or 3-vessel disease vs none or 1-vessel disease (yes or no) [6100, 6110, 6120, 6130, 6140, 6150, 6160]	Existing full model
Lesion complexity	High complexity or nonlesion (yes or no) [7185]	NA
SCAI lesion class	I, II, III, or IV [7185, 7115]	NA
SCAI lesion class: II or III	SCAI lesion is class II or III (yes or no) [7185, 7115]	Existing full model
SCAI lesion class: IV	SCAI lesion is class IV (yes or no) [7185, 7115]	Existing full model
CAD presentation	CAD presentation [5000]	NA
STEMI	If CAD presentation is STEMI (yes or no) [5000]	Existing simplified risk score, existing full model
Stenosis % before treatment	% Stenosis immediately before treatment [7115]	NA
Subacute stent thrombosis	In-stent thrombosis [7145, 7150, 7165]	Existing full model
**Characteristics and PCI Status at Presentation**		
PCI status and shock composite	1: Salvage and shock (within 24 h and at start of PCI)	NA
	2: Salvage or shock (within 24 h and at start of PCI)	
	3: Shock within 24 h or at start of PCI	
	4: Emergent procedure	
	5: Urgent procedure	
	6: Elective procedure [5060, 7020, 7030]	
PCI status and shock composite 1	Salvage and shock (within 24 h and at start of PCI) (yes or no) [5060, 7020, 7030]	NA
PCI status and shock composite 2	Salvage or shock (within 24 h and at start of PCI) (yes or no) [5060, 7020, 7030]	Existing full model
PCI status and shock composite 3	Shock within 24 h or at start of PCI (yes or no) [5060, 7020, 7030]	Existing full model
PCI status and shock composite 4	Emergent procedure (yes or no) [5060, 7020, 7030]	Existing full model
PCI status and shock composite 5	Urgent procedure (yes or no) [5060, 7020, 7030]	Existing full model
PCI status and shock composite 6	Elective procedure (yes or no) [5060, 7020, 7030]	NA
Shock within 24 h and at start of PCI	Shock within 24 h and at start of PCI (yes or no) [5060, 7030]	Existing full model
Lytics before PCI for STEMI	Lytics before PCI for STEMI (yes or no) [5000, 5010]	Existing full model
Cardiac arrest within 24 h	Cardiac arrest within 24 h (yes or no) [5065]	Existing simplified risk score, existing full model
Cardiogenic shock within 24 h	Cardiogenic shock within 24 h (yes or no) [5060]	NA
Cardiogenic shock within 24 h or at start of PCI	Cardiogenic shock within 24 h or at start of PCI (yes or no) [5060, 7030]	Existing simplified risk score
Cardiogenic shock composite	1: Cardiogenic shock within 24 h and at start of PCI	NA
	2: Cardiogenic shock within 24 h	
	3: Cardiogenic shock at start of PCI	
	4: No cardiogenic shock [5060, 7030]	
Cardiogenic shock at start of PCI	Cardiogenic shock at start of PCI [7030]	NA
PCI status composite	1: Elective	Existing simplified risk score
	2: Urgent	
	3: Emergency	
	4: Salvage [7020]	
**Laboratory Values**		
Preprocedural hemoglobin	Preprocedural hemoglobin (continuous) [7320]	Existing full model
Preprocedural hemoglobin ≤13 g/dL	Preprocedural hemoglobin ≤13 g/dL (yes or no) [7320]	Existing full model
Preprocedural hemoglobin >13 g/dL	Preprocedural hemoglobin >13 g/dL (yes or no) [7320]	NA
Preprocedural LVEF %	Preprocedural LVEF % (continuous) [7025]	Existing simplified risk score
Preprocedural creatinine	Preprocedural creatinine (continuous) [7315]	NA

Abbreviations: BMI, body mass index (calculated as weight in kilograms divided by height in meters squared); CAD, coronary artery disease; GFR, glomerular filtration rate; LAD, left anterior descending; LVEF, left ventricular ejection fraction; NA, not applicable (additions to the blended model data set); NCDR, National Cardiovascular Data Registry; NYHA, New York Heart Association; PCI, percutaneous coronary intervention; SCAI, Society for Cardiovascular Angiography and Interventions; STEMI, ST-segment elevation myocardial infarction; TIMI, thrombolysis in myocardial infarction.

SI conversion factor: To convert hemoglobin level to grams per liter, multiply by 10.0.

### Experiment 1: Machine Learning Techniques and the Existing Simplified Risk Score

Results for the existing simplified risk score and the machine learning techniques are summarized in Table 3. The existing simplified risk score achieved a mean C statistic of 0.77 (95% CI, 0.77-0.77), similar to the 0.76 reported by Rao et al^3^ after excluding CABG cases. Adding lasso regularization did not alter the model’s discrimination. Using the same 10 variables, XGBoost improved discrimination of the mean C statistic to 0.81 (95% CI, 0.80-0.81).

**Table 3.** C Statistics of 5-Fold Cross-validation Results for the Existing Simplified Risk Score and the Blended Model

Timing	Variable Set	Mean (95% CI) C Statistic
Existing simplified risk score	Existing simplified risk score	0.77 (0.77-0.77)
	Existing simplified risk score with lasso regularization	0.77 (0.77-0.77)
	Existing simplified risk score with gradient descent boosting	0.81 (0.80-0.81)
Blended model	Existing full model	0.78 (0.78-0.78)
	Existing full model with lasso regularization	0.78 (0.78-0.78)
	Existing full model with gradient descent boosting	0.78 (0.78-0.78)
	Blended model with lasso regularization	0.78 (0.78-0.78)
	Blended model with gradient descent boosting	0.82 (0.82-0.82)

Table 4 lists the prediction results of the existing simplified risk score variable set and the existing simplified risk score variable set with XGBoost. Using the existing simplified risk score threshold for high risk of 65.0 points (6.5% risk),^3^ we correctly identified 105 316 cases as high risk for bleeding who experienced an event and 2 208 569 cases as low risk for bleeding who did not experience an event. However, we classified 44 408 cases as low risk for bleeding who experienced an event and identified 958 172 cases as high risk for bleeding who did not experience an event, yielding a false discovery rate of 90.1% and a positive predictive value of 9.9%. Using a data-driven threshold of 96.4 points (between 14.9% and 17.0% risk) correctly identified 47 445 bleeding cases and 2 990 509 nonbleeding cases (21.8% of cases, or 21 833 corrected cases per 100 000 PCI cases), yielding a false discovery rate of 78.8% and a positive predictive value of 21.2%. The 10-variable existing simplified risk score variable set modeled with XGBoost, selecting a data-driven risk threshold of 15.1%, correctly identified 52 768 cases as high risk for bleed who experienced an event and 3 019 006 cases as low risk for bleeding who did not experience an event (22.9% of cases, or 22 852 corrected cases per 100 000 PCI cases) and a false discovery rate of 73.7%.

**Table 4.** Prospective Predictions and Changes in 5-Fold Cross-validation for the Existing Simplified Risk Score and the Blended Model^a^

Timing	Variable Set	Prospective Prediction				Mean (SD) Decision Threshold
		True-Positives	True-Negatives	False-Positives	False-Negatives	
Existing simplified risk score	Existing simplified risk score with high-risk threshold	105 316	2 208 569	958 172	44 408	65.0 (0.0) Points (6.5%)
	Existing simplified risk score with data-driven threshold	47 445	2 990 509	176 232	102 279	96.4 (0.1) Points (range, 14.9%-17.0%)
	Existing simplified risk score with lasso regularization	52 420	2 954 631	212 110	97 304	10.0% (0.3%)
	Existing simplified risk score with gradient descent boosting	52 768	3 019 006	147 735	96 956	15.1% (0.8%)
Blended model	Existing full model	49 967	2 982 389	184 352	99 757	11.9% (0.5%)
	Blended model with lasso regularization	51 840	2 977 168	189 573	97 884	11.6% (0.4%)
	Blended model with gradient descent boosting	55 527	3 013 868	152 873	94 197	15.6% (0.9%)

^a^ The “NA” fields in Table 2 are the baseline models and have no previous method against which to compare change.

### Experiment 2: Machine Learning Techniques and the Existing Full Model

Results for the existing full model and the machine learning techniques are summarized in Table 3. The existing full model achieved a mean C statistic of 0.78 (95% CI, 0.78-0.78), similar to the 0.78 reported by Rao et al^3^ after excluding CABG cases. Neither lasso regularization nor XGBoost improved model discrimination.

### Experiment 3: Machine Learning Techniques and the Blended Model

Results for the blended model variable set are summarized in Table 3 and eTable 1 in the Supplement. Logistic regression with lasso regularization achieved a mean C statistic of 0.78 (95% CI, 0.78-0.78). XGBoost improved model discrimination to a mean C statistic of 0.82 (95% CI, 0.82-0.82). The blended model had an *F* score of 0.31 (95% CI, 0.30-0.31) vs 0.26 (95% CI, 0.26-0.26) for the existing full model.

Table 4 summarizes the progression of model improvement, from the existing full model variable set using logistic regression and the blended model NCDR bleeding risk variable set using logistic regression, to the blended model variable set modeled with XGBoost. The existing full model, with a data-driven risk threshold of 11.9%, correctly identified 49 967 cases as high risk for bleeding who experienced an event and 2 982 389 cases as low risk for bleeding who did not experience an event, yielding a false discovery rate of 78.7%. The blended model trained with logistic regression, at a data-driven risk threshold of 11.6%, correctly identified 51 840 cases as high risk for bleeding who experienced an event and 2 977 168 cases as low risk for bleeding who did not experience an event, an increase in 1873 cases identified as high risk for bleeding who experienced an event but a decrease in 5221 cases identified as low risk for bleeding who did not experience an event, yielding a false discovery rate of 78.5%. The blended model trained with XGBoost, at a data-driven risk threshold of 15.6%, correctly identified 55 527 cases as high risk for bleeding who experienced an event and 3 013 868 cases as low risk for bleeding who did not experience an event, an increase in 5560 cases identified as high risk for bleeding who experienced an event (3.7% of bleeding cases, or an additional 168 bleeding cases per 100 000 PCI cases) and 31 479 nonbleeding cases (1.0% of nonbleeding cases, or an additional 949 nonbleeding cases per 100 000 PCI cases), yielding a false discovery rate of 73.4%.

### Experiment 4: Understanding the Blended Model

Calibration plots are shown for the existing full model and the blended model divided into deciles, with associated standard errors (Figure, A). Also shown are continuous calibration plot functions of risk and 95% CIs (Figure, B). The blended model demonstrated a closer calibration than the existing full model, with a Brier score of 0.039 vs 0.041. eFigures 2, 3, 4, and 5 in the Supplement show the improvement in predictions in the highest deciles of risk (eAppendix in the Supplement).

**Figure.**
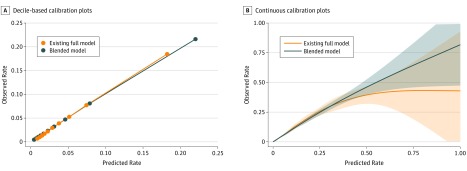
Plots for the Existing Full Model and the Blended Model The blended model demonstrated a closer calibration than the existing full model. A, Decile-based calibration plots are calculated from the 5-fold cross-validation showing stable model calibration. B, Continuous calibration plots with 95% CIs (shaded areas) are shown for the 2 models.

eTables 2, 3, and 4 in the Supplement show the variables for the blended model, along with the forward stepwise selection C statistic when added in rank order to the model. Using only the top 10 predictive variables achieved a mean C statistic of 0.81 (eAppendix in the Supplement).

## Discussion

Our study shows that machine learning algorithms better characterize the risk of major bleeding after PCI. However, the ability of machine learning techniques to produce better results depends on whether extracted variables are constrained. Our results comparing logistic regression with lasso regularization against XGBoost with the predefined, dichotomous variables show that implementing machine learning algorithms does not necessarily improve predictive results. By not constraining available data to dichotomous variables before the modeling and then leveraging machine learning techniques, we observed improvements in models. These models were better able to identify high-risk individuals and reclassified the risk of a meaningful percentage of patients. Therefore, models improved with machine learning techniques, but processing of data has a large role in effectiveness. Such models have the possibility of being integrated into electronic medical records and made available at the point of care.

Using machine learning techniques, we improved the discrimination of the existing full model of bleeding risk. By using the data-driven thresholds to optimize decision making, we correctly classified an additional 5560 cases as high risk for bleeding who experienced an event and 31 479 cases as low risk for bleeding who did not experience an event (an additional 168 bleeding cases per 100 000 PCI cases and an additional 949 nonbleeding cases per 100 000 PCI cases, respectively). Similarly, the machine learning techniques model improved the discrimination of the existing simplified risk score model to 0.82 from 0.78 with no additional variables because gradient descent boosting is better equipped to fully analyze continuous and categorical variable ranges. This work demonstrates an improvement in model discrimination and calibration and includes an evaluation of the ability of models to identify specific bleeding cases and nonbleeding cases; the improved identification of both bleeding cases and nonbleeding cases is ultimately necessary to address how clinicians could use risk scores in real time.

Nevertheless, prospective application of machine learning techniques requires more research. Other work (eg, that by Spertus et al^21^ and from an associated product called Patient Risk Information Services Manager [ePRISM]) attempts to link outcomes research, clinical decision making, and informed consent. We believe such work demonstrates a path forward for using a model for quality improvement, consent (as in the work by Spertus et al^21^), and additional risk mitigation on a prospective case-by-case basis. We chose a data-driven selection of the risk threshold to equalize misclassification errors. If the treatment for FP results and for FN results is found to differ in costs, time associated with treating them, and other important resources, the decision threshold may require adjustment.

The predictive enhancements demonstrated herein have 2 implications. The first involves variable selection, whereby thresholds that emerge from the data allow for the use and interpretation of continuous values rather than forcing preselection of dichotomous thresholds. For example, XGBoost analysis of pre-PCI hemoglobin as a continuous value rather than the dichotomous threshold (≤13 vs >13 g/dL) at a minimum reduces preprocessing efforts and potentially enables further insight into what the critical values are in predicting risk for patients. A second implication is monitoring of higher-order interactions. By finding potentially nonlinear combinations of values, a machine learning model can better characterize risk.

### Limitations and Future Work

Our study has some limitations. First, we used a simple data imputation strategy due to the low rate of missing values in the data set. While this may introduce some faulty performance, our improved results will become stronger with advanced, patient-specific imputation techniques that should be further investigated. Second, despite the large number of variables we incorporated, we could not adjust for several other factors that may augment risk prediction. For example, we were unable to adjust for the access-site decision; to keep a strict comparison of modeling techniques possible, we did not include this information. Such a decision would result in downstream differences in treatments. We are now able to evaluate the influence the additional variables collected in the registry will have on risk, particularly in the extreme low-risk and high-risk scenarios with access site and anticoagulation decisions.

Evaluating the predictive differences in the existing simplified risk score model vs the blended model (which contains periprocedural variables) shows variations in prediction that identify changes in patient risk throughout the course of treatment. The development of models that include additional variables and decisions made during treatment would require multiple, staged models to identify patient risk. This would aid clinical decision making in elucidating the dynamic factors that change risk, understanding what variables are readily available in electronic health records for tool development, and further discussing the clinical implementations of such models. In addition, comparing results with the different potential types of bleeding may clarify the difference in FP and TP results. For example, the definition of major bleeding includes a hemoglobin decrease that may not actually correspond to major bleeding that was identified and treated. Similarly, other values may indicate major bleeding that does not fit the definitions. A further exploration of the misclassifications and their causes may provide additional clinical value.

## Conclusions

Using machine learning methods strategically allows for improvements in predictive model performance. Knowing the data ranges measured and how data fit into machine learning techniques enables us to realize the potential of these techniques. When applied with an appropriate variable set, machine learning techniques improved risk prediction models for major bleeding after PCI. We demonstrated that machine learning techniques will not necessarily do the work of improving predictive value and that a key to successful implementation is the use of variables in a way that does not reduce information. We showed that the application of these methods improved model discrimination (C statistic, 0.82) and calibration and offered direct metrics of how the model would perform with a prospective cohort of patients (*F* score, 0.31). These findings lay the groundwork for future work in more advanced models with additional variables for further improved performance.

## Notes

Supplement. **eFigure 1.** Patient Inclusion and Exclusion Criteria **eFigure 2.** Difference in Risk Prediction Between the NCDR Existing Full Model and the Blended Model **eFigure 3.** The Quantity of Bleeds Correctly Identified by the Blended Model **eFigure 4.** The Quantity of Non-bleeds Correctly Identified by the Blended Model **eFigure 5.** The False Discovery Rate and Positive Predictive Value of the Blended Model **eAppendix.** Explaining Implementations of Boosted Decision Trees and Additional Variable Selection Validation Strategies **eTable 1.** Model Results for Patient Cohorts **eTable 2.** Blended XGBoost Model Incremental Variable Importance **eTable 3.** Blended Model Dataset Features; Average Rank in 5-Fold Cross-validation and Times in Top 10 **eTable 4.** Blended Model Dataset Features; Stepwise Forward Selection C-statistic in 90/10 Training/Testing Split
