# Supplementary material for: Comparison of Machine Learning Methods With National Cardiovascular Data Registry Models for Prediction of Risk of Bleeding After Percutaneous Coronary Intervention
Source: JAMA Netw Open. 2019 Jul 10;2(7):e196835. doi: 10.1001/jamanetworkopen.2019.6835 (PMC6624806; doi:10.1001/jamanetworkopen.2019.6835)
Supplement: Supplement. — eFigure 1. Patient Inclusion and Exclusion Criteria eFigure 2. Difference in Risk Prediction Between the NCDR Existing Full Model and the Blended Model eFigure 3. The Quantity of Bleeds Correctly Identified by the Blended Model eFigure 4. The Quantity of Non-bleeds Correctly Identified by the Blended Model eFigure 5. The False Discovery Rate and Positive Predictive Value of the Blended Model eAppendix. Explaining Implementations of Boosted Decision Trees and Additional Variable Selection Validation Strategies eTable 1. Model Results for Patient Cohorts eTable 2. Blended XGBoost Model Incremental Variable Importance eTable 3. Blended Model Dataset Features; Average Rank in 5-Fold Cross-validation and Times in Top 10 eTable 4. Blended Model Dataset Features; Stepwise Forward Selection C-statistic in 90/10 Training/Testing Split [file jamanetwopen-2-e196835-s001.pdf]

Supplementary Online Content

Mortazavi BJ, Bucholz EM, Desai NR, et al. Comparison of machine learning methods with National Cardiovascular Data Registry models for prediction of risk of bleeding after percutaneous coronary intervention. *JAMA Netw Open*. 2019:2(7):e196835. doi:10.1001/jamanetworkopen.2019.6835

eFigure 1. Patient Inclusion and Exclusion Criteria

eFigure 2. Difference in Risk Prediction Between the NCDR Existing Full Model and the Blended Model

eFigure 3. The Quantity of Bleeds Correctly Identified by the Blended Model

eFigure 4. The Quantity of Non-bleeds Correctly Identified by the Blended Model

eFigure 5. The False Discovery Rate and Positive Predictive Value of the Blended Model

eAppendix. Explaining Implementations of Boosted Decision Trees and Additional Variable Selection Validation Strategies

eTable 1. Model Results for Patient Cohorts

eTable 2. Blended XGBoost Model Incremental Variable Importance

eTable 3. Blended Model Dataset Features; Average Rank in 5-Fold Cross-validation and Times in Top 10

eTable 4. Blended Model Dataset Features; Stepwise Forward Selection C-statistic in 90/10 Training/Testing Split

This supplementary material has been provided by the authors to give readers additional information about their work.

© 2019 Mortazavi BJ et al. *JAMA Network Open*.

eFigure 1. Patient inclusion and exclusion criteria.

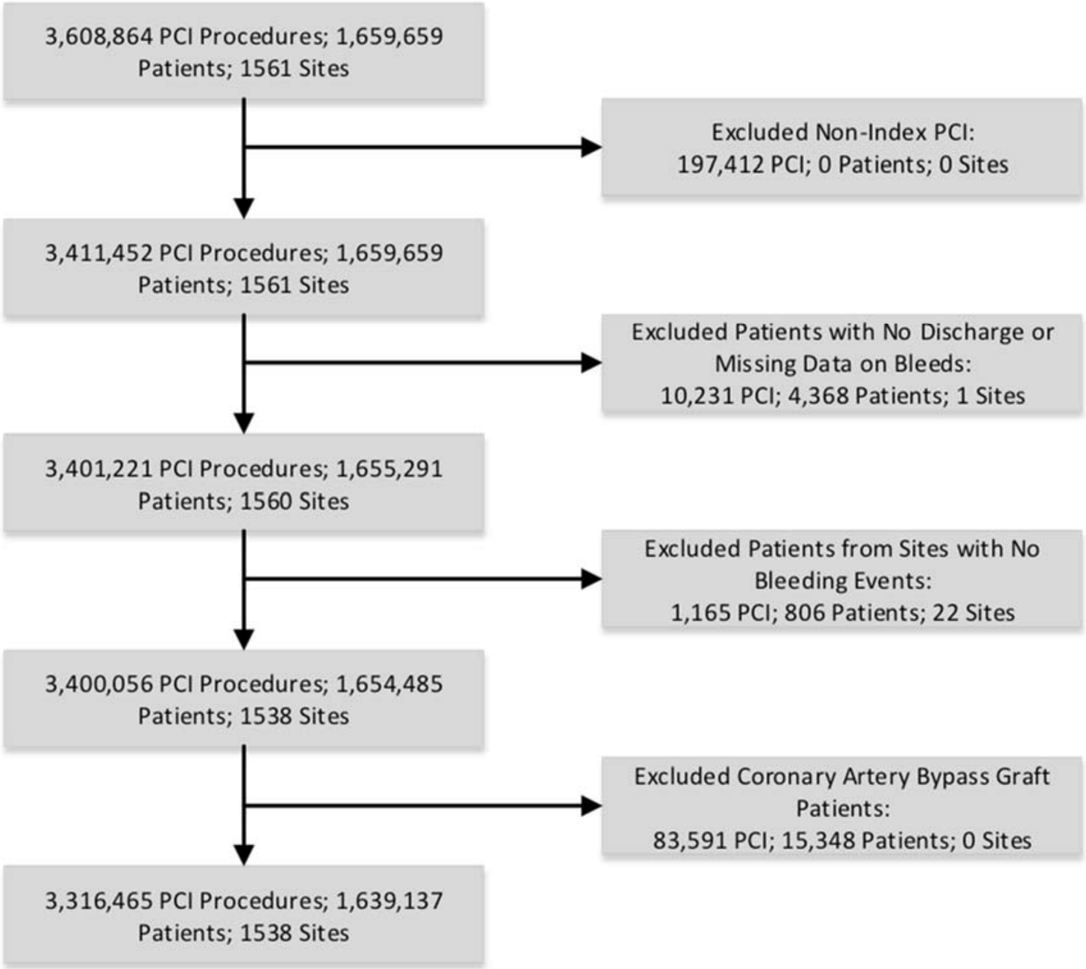

© 2019 Mortazavi BJ et al. *JAMA Network Open*.

eFigure 2. Difference in risk prediction between the NCDR existing full model and the blended model.

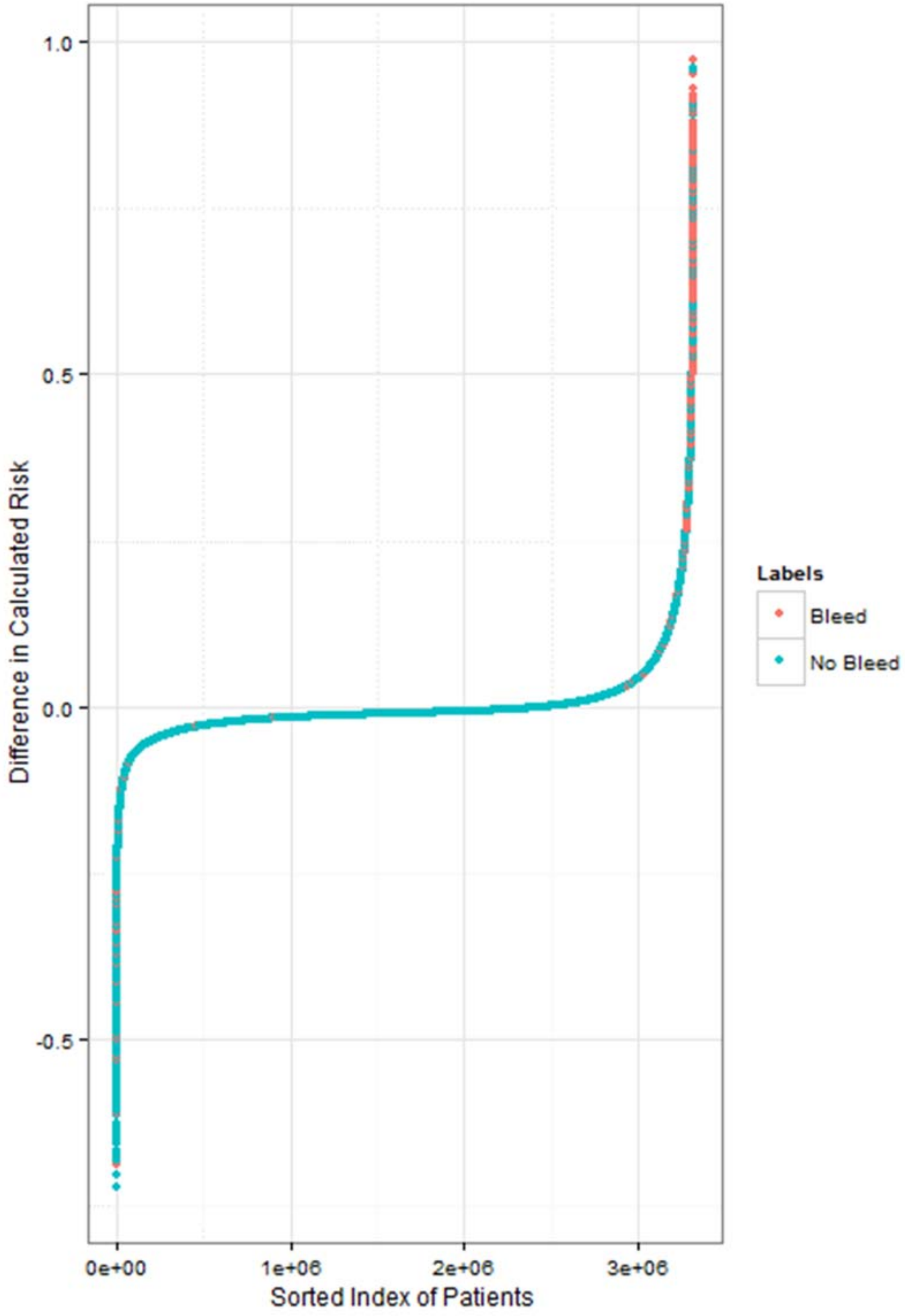

© 2019 Mortazavi BJ et al. *JAMA Network Open*.

eFigure 3. The quantity of bleeds correctly identified by the Blended model. (Quantity of bleeds identified by the Blended model when considering the (a) highest decile of risk as a decision threshold and (b) mean predicted risk of the highest decile as a decision threshold.)

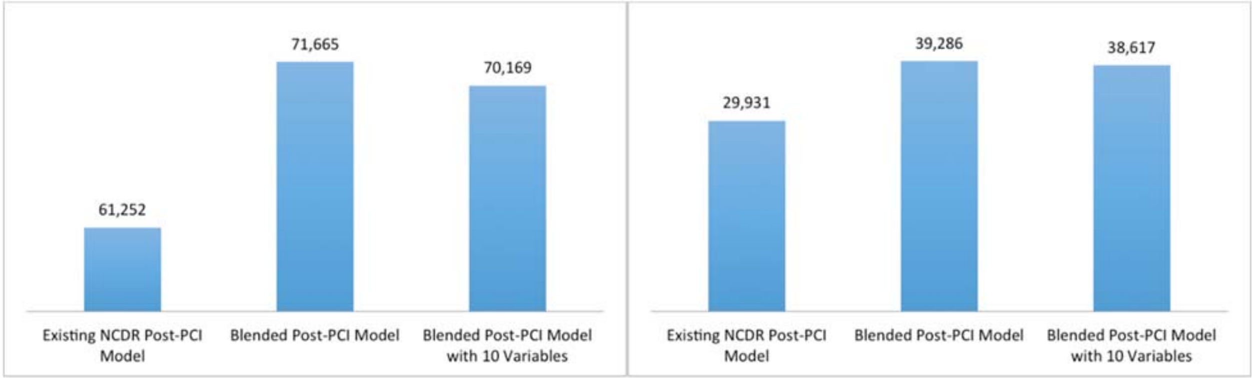

NCDR = National Cardiovascular Data Registry; PCI = percutaneous coronary intervention

eFigure 4. The quantity of non-bleeds correctly identified by the Blended model. (Quantity of bleeds identified by the Blended model when considering the (a) highest decile of risk as a decision threshold and (b) mean predicted risk of the highest decile as a decision threshold.)

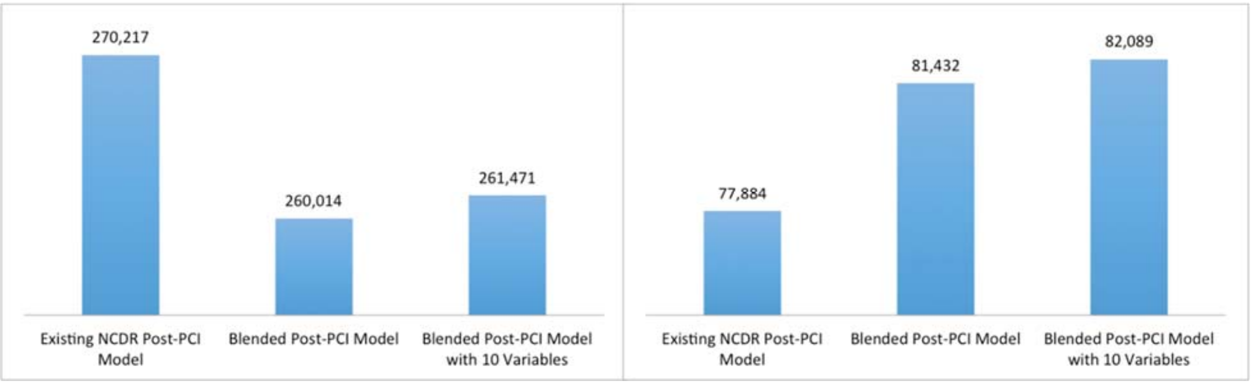

NCDR = National Cardiovascular Data Registry; PCI = percutaneous coronary intervention

eFigure 5. The false discovery rate and positive predictive value of the Blended model. (False discovery rates and positive predictive values of the Blended model when considering the (a) highest decile of risk as a decision threshold and (b) mean predicted risk of the highest decile as a decision threshold.)

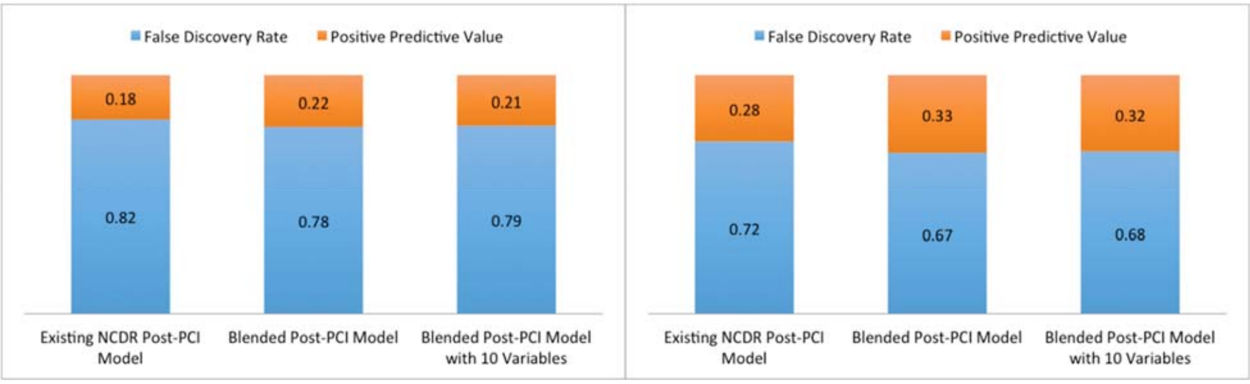

NCDR = National Cardiovascular Data Registry; PCI = percutaneous coronary intervention

© 2019 Mortazavi BJ et al. *JAMA Network Open*.

eAppendix. Explaining Implementations of Boosted Decision Trees and Additional Variable Selection Validation Strategies

I. Motivation

We sought to improve prediction of bleeding using machine learning methods compared with an existing model that was derived from the same dataset. With the machine learning methods, we started with variables that had been selected or defined in the existing model, and then extended this set to any additional variables related to those variables selected or defined in the existing model (e.g., pre-procedure hemoglobin continuous value rather than 2 variables of pre-procedure hemoglobin ≤13 and >13 g/dL). We did conduct additional experiments to determine if any improved performance from the machine learning models was a result of new variable selection or the use of a different analytic approach. Finally, we conducted supplementary analyses to determine how effective the machine learning models would be if they selected a smaller set of the most predictive variables, so that this targeted set could be more acquired for incorporation into post-PCI clinical care and decision making.

For this reason we evaluated two different methods. The first is a traditional statistical technique in logistic regression, but with lasso regularization, to understand if a difference in the method by which logistic regression selects the variables impacts performance. The second was gradient descent boosting, which is better equipped to develop models that have binary, categorical, and continuous data. We conducted analyses to determine if the variables provided the greatest impact, the methodology, or the combination of variables and methodology, while still using techniques that maintained a level of interpretability for clinical understanding.

II. Inclusion Criteria and Outcomes Definitions

Our initial sample used inclusion and exclusion criteria for the existing full NCDR bleeding-risk model,[1] updated to include all index PCI procedures from July 2009 through April 2015 (**eFigure 1**). Briefly, this study population excluded patients who had repeated PCI procedures per admission (197,412 cases), died in the hospital or had missing bleeding information (10,231 cases), or from sites with no bleeding events (1,165 PCI cases from 22 sites). We also excluded patients who underwent coronary artery bypass grafting (CABG) during the index admission, because the high risk of bleeding after CABG may obscure the bleeding risk attributable to PCI alone.[2] We must note a limitation in our work regarding de-identified procedure admission information. Namely, because our dataset is de-identified, we can only identify PCI procedures that are related if they occur in the same admission. Therefore, the distinct number of procedures we have isolated do not necessarily mean each is a new patient. This has the potential to introduce some bias through patients with repeated admissions and PCI, and is a limitation of our work.

The primary outcome, as in the existing NCDR bleeding model, was major post-PCI bleeding. The outcomes definitions are identical to those in work by Rao et al.[3] Major bleeds are: 1) Major bleeding occurring within 72 hours after the PCI or before discharge is said to have occurred if there is a site-reported bleed (external or hematoma >10 cm, >5 cm, and >2 cm for femoral, brachial, and radial access, respectively); 2) Bleeds reflecting a post-PCI hemoglobin decrease of 3 g/dL in patients with a pre-PCI level of at least 16 g/dL; 3) Any non-surgery blood transfusion for pre-procedure hemoglobin levels of at least 8 g/dL; 4) intracranial hemorrhage, cardiac tamponade, and gastrointestinal or retroperitoneal bleeding.[3]

© 2019 Mortazavi BJ et al. *JAMA Network Open*.

III. Extreme Gradient Boosting (xgboost)

Gradient descent boosting was selected as the primary machine learning technique used for this analysis for several reasons. First, decision tree-based methods are inherently more interpretable than popular deep learning techniques. By seeing how often variables are selected across a variety of the decision trees made, we are able to interpret how important each variable is. Second, decision tree-based methods are able to make use of multimodal data seamlessly. In other words, they are able develop models that have binary variables, categorical variables, and continuous variables alike. Finally, with regards to the variety of decision tree-based methods that exist, we chose xgboost because of the advantages this technique provides. This method develops one decision tree at a time that has limited depth. This requires this tree to find variables that best split the population, in the hopes of having leaf nodes at the bottom of the tree that best split bleeding cases versus. non-bleeding cases. After this tree is developed, through a series of tests that identify the best variable for this split (accounting for potential outliers in the data), the model determines how much of the training set variation can be explained by this tree. Based upon this it develops the next tree with the inherent goal of better explaining the variation in the proportion of the training set not explained by the first decision tree. This procedure continues until a group of trees helps develop a robust predictive model. For more details we encourage readers to understand why this technique is potentially preferable to other decision tree techniques such as random forest, as explained by the authors of the technique.[4]

When the trees are finished training, our predictive model has several interpretable factors. The top variables selected across each of the trees indicates important variables in understanding low versus high risk procedure cases. Second, the higher a variable is on the tree the more important it was deemed to be in understanding bleeding cases versus non-bleeding cases. Finally, we can understand risk for each individual patient by understanding the paths in each tree that predicted that patient's risk.

IV. Cross-Validation and Model Hyper Parameters

The cross-validation process described in the main text for the new models was also repeated for the 2 existing NCDR bleeding-risk models to detect any differences in discrimination that might arise from using a cross-validation approach rather than the single-derivation/validation cohort split used in prior work.[1] This allowed us to directly compare performance of the existing technique in this stratified cross-validation approach to the new methods and variables considered.

All analyses were conducted in R, with the base GLM function used for logistic regression with the pre-selected variables to recreate the existing models, the GLMNET package used for the logistic regression with lasso regularization,[5] XGBOOST for the gradient descent boosting,[4] and pROC for the ROC and c-statistic calculations.[6] We used mgcv and sandwich for the continuous smoothing functions for calibration curves.[7,8]

For the GLMNET package in R, the hyper parameters for the model were set by using the default values in the cv.glmnet function with a 10-fold internal cross-validation. This is pre-built in the package glmnet (cv.glment). This method creates, from the training set, an internal 10-fold training and testing set. It iterates through and compares different tuning parameters and selects the lambda that provides the highest AUROC within the training. This lambda is learned from

the training set (along with the other parameters) and is used as the default for prediction in the testing set.

For xgboost, we set the number of trees to 1000, with an eta of 0.1 and a maximum depth of each tree of 6. We used the default learning rate and depth of tree, and preset the number of trees for computational efficiency but to provide a sufficient number of trees since boosting learns slowly. This limitation should be addressed in future work to grid search a wider number of trees (100 to 10000) with varying depth (from 1 to 10).

V. Implementation

In order to implement these methods the data must be taken through several pre-processing steps. First, the cohort must be extracted, as in II above. Then the data must be split for internal validation. In our case we take the process of randomly separating 80 % of the data for training, and holding out 20% for validation, while keeping the event rate consistent in that. At this point we run imputation techniques based on the 80% training data. We then feed this training data and the training labels to the machine learning methods. In the case of xgboost we discuss the parameters used to tune the model in the prior section (IV). Finally, we then generate a prediction on the final 20%. We repeat this process five times, with a new 20% of the data serving as the test case every iteration. In R, this amounts to three lines of code. The first train the xgboost model with the data matrix where each procedure is a row and each column is a variable, we then generate a prediction using the model and the test data, we finally compare that prediction with the ground truth for the test set in pROC to plot the ROC curve. Source code is available at https://github.com/bobakm/NCDR_CathPCI_MajorBleed_Public.

VI. Using the final model

In order to recreate these models for use, we have made our source code available to extract the same patient cohort if one has access to the CathPCI registry data. The specific variables and hyperparameters are provided to the training of the xgboost model. For using the model, new test cases will have missing data imputed based upon the training set, and as described in the body of the paper. This model will produce a probability associated with the risk of major bleeding post-PCI.

VII. Additional Dataset Comparisons

We sought to better understand the updated samples. Specifically, we split analyses by year, for cases considered in the existing NCDR bleeding-risk models and newly collected cases, to confirm that changes in bleeding rates did not affect model discrimination (they did not) and that we are recreating the performance of the existing technique. Additionally, we provided supplementary analyses to ensure our top features selection technique was a fair selector of variables.

The datasets used added variables in specific orders to determine the impact of variables versus methods. The blended variables set had 28 additional variables that primarily included continuous-variable versions of variables that the existing model had converted to dichotomous variables; continuous variables such as pre-procedural hemoglobin, previously used as 2 dichotomous variables of pre-procedural hemoglobin (≤13 and >13 g/dL), were also added to the dataset. This variable set will provide the best performing model. Methods will be compared in this set to understand the model improvement resulting from method as well as resulting from the additional 28 variables. The post-PCI variable set was used to provide a direct comparison to

the existing technique to evaluate improvements as a result of machine learning techniques specifically. The pre-PCI variable set was created to evaluate impact of the risk score, which uses different decision thresholds than the post-PCI model, the continuous version of these variables was used for this dataset. This variable set provided a direct comparison to the existing risk score technique to evaluate improvements as a result of machine learning techniques specifically for data-driven decision thresholds.

To verify that the additional samples did not affect discrimination, we compared data available to us from July 2009 to April 2011, similar to the data range of the existing NCDR bleeding model, which had an event rate of 4.6%, and from all the subsequent additional observations (May 2011 to April 2015) that had an event rate of 4.9%. The c-statistic for the additional samples, using the existing NCDR bleeding model in a 5-fold cross-validation, was 0.78 (0.77-0.78). This c-statistic was similar to the existing NCDR bleeding model on the original sample.

Additionally, we compared the final blended model to that of the blended model, using only 10 variables for a variety of reasons. First, the pre-PCI model also had 10 variables. These variables could be used to replace the pre-PCI case. Second, the eleventh variable (cardiogenic shock within 24 hours) is co-linear to the ninth variable (cardiogenic shock within 24 hours or at the start of PCI).

VIII. Feature Selection and Ranking

The feature selection and ranking techniques for xgboost are detailed in the manuscript. The full xgboost ranking of selected features can be found in **eTable 2,** which includes not only the ranked features from the entire dataset but also those features not selected by the model. However, as mentioned in the main manuscript, the ranking of the top 10 variables, and their contribution to the forward selected c-statistic, could be considered an unfair comparison. In particular, the dataset is trained on the entire data, and we can assume the model is a good fit by the 5-fold cross-validation. However, the c-statistic calculated by the forward selection is a result of using training data and testing data together, which is not ideal. We wanted to develop a technique that identifies the top contributors. To show that the results are a fair indicator of the results, we ran several other feature-ranking techniques.

Using the blended dataset, we ensured that the top-10 comparisons were fair by validating them in several different tests. First, in a 5-fold cross-validation using the training data as the testing data, we ensured that the model is not extremely overfitting. Second, we ensured that the top-10 variables are stable across each fold and that each top-10 variable was in the final top 10 of multiple folds, and ensured the consistency from each fold to the total dataset by showing the average ranking of each feature and its standard deviation across the 5 folds. Third, we ensured that the stepwise feature selection was fair by showing a stepwise selection with a 90/10 training/testing split that had similar incremental gains.

By running the 5-fold cross-validation on the blended dataset again, we can check off the first 2 tests together. First, the mean c-statistic (and 95% confidence interval) for using the training data as testing data in each fold was 0.838 (0.838-0.839), higher than the 0.82 achieved when using the testing set. This upper bound on the c-statistic is similar to the 0.82 achieved with a test set. This means using the entire training set to determine feature importance cannot alter the stepwise results by a large margin. **eTable 3** uses the variables (in rank order from the main manuscript) to show their average feature rank and number of times they appear in the top 10 in each fold in the 5-fold cross-validation. The average ranking and low standard deviation show

the stability of the selection of the top 10 variables. Finally, **eTable 4** shows the forward stepwise c-statistic calculation for the top-10 variables ranked in a more traditional fashion: showing the variables selected in a 90% training dataset and then tested in a forward stepwise fashion on the remaining 10% testing data. The values show incremental improvements very similar to those listed in the manuscript.

IX. Additional Results on Decision Thresholds

The evaluation of the decision threshold and the model calibration give an evaluation of the model's performance when used prospectively to decide whether a patient should receive bleeding-avoidance therapies, and to evaluate our performance via the f-score, positive predictive value, and false discovery rate (ratio of false positives to all positive predictions). **eTable 1** shows the f-score for the best model in each variable set. The existing pre-PCI NCDR bleeding risk model achieved a mean f-score of 0.25 (0.25-0.26) and the best model in each variable set of post-PCI NCDR model. The existing post-PCI NCDR bleeding-risk model achieved a mean f-score of 0.26 (0.26-0.26), which did not change when switching between modeling methods.

X. Additional Evaluations of Risk

We intended to show that risk is somewhat dynamic, and understand the difference between the bedside risk score and the full risk model. **eFigure 2** takes each patient's risk, as calculated by the best performing model using the 10 variables from the pre-PCI model, and the risk calculated by the best performing full post-PCI model, and takes the difference. The clustering of bleeds towards the positive nature shows that the full post-PCI model raises the risk score of a lot of patients, most of whom have a bleeding outcome. This visually demonstrates an improvement in the c-statistic, as well as indicate more evidence that a specific threshold can separate bleeds and non-bleeds. **eFigure 2** shows the risk difference when calculating the bleeding risk from the blended variable set and from the existing post-PCI NCDR bleeding-risk model. Overall, the model trained on the blended variable set more accurately identified post-procedural bleeding risk over the model trained on the existing NCDR bleeding-risk variable set, evidenced by the higher concentration of bleeds in the region of largest difference in risk between the blended model and the existing post-PCI NCDR bleeding-risk model (right side, **eFigure 3**).

Additionally, we evaluated the performance of our models with a variety of decision thresholds. Specifically, in order to develop an ROC curve, a decision threshold is varied between a probability of 0 and a probability of 1. At each of these evaluation points, it is possible to calculate decision threshold-specific metrics. We ultimately present the threshold-specific metrics based upon the threshold that results in the highest f-score. The balance used in this study assumes an equal cost between false positives and false negatives. However, this may not be the case. Certain institutions may wish to use bleeding avoidance medications on patients with minimal risk, while others may wish to treat different risk thresholds with different strategies. In order to evaluate this performance, we also compared the positive predictive value and false discovery rates of the methods by using the highest decile of risk, rather than the data driven threshold, to show that the performance gains still exist. **eFigures 3 and 4** show the quantity of bleeds and non-bleeds identified when selecting a threshold at the decile boundary and at the mean rate of the decile. Quantities, however, might be misleading due to the number of people at or above the mean rate of the decile versus in the decile entirely, so we show the rates in **eFigure**

5. We see the false discovery rate drops when using the full post-PCI model trained by xgboost, and that the parsimonious model using the top predictors performs similarly well.

Since the calibration plot identifies the largest difference in the highest decile, we further analyzed the predictive nature of the model in this decile. **eFigure 4** shows the correctly identified bleeds when using the decile threshold and mean decile rate, respectively. The highest decile of risk is any predicted risk ≥9.5% for the existing NCDR post-PCI bleeding-risk model, 10.9% for the blended post-PCI model, and 10.8% for the blended post-PCI bleeding model using only the top-10 predictive variables. The mean predicted rate for the highest decile of risk was 18.2% for the existing NCDR post-PCI bleeding risk model, 22.0% for the blended post-PCI model, and 21.5% for the blended post-PCI model with 10 variables. **eFigure 4** shows the incorrectly treated non-bleeds**.** While the FPs drop greatly when viewing the highest decile of risk and using the blended model, the quantity of FPs increases when using the mean predicted risk as a decision threshold. Note that the optimally selected thresholds in **Table 4** are between these 2 rates. However, fewer cases are at a level of risk at or above the mean in the existing NCDR post-PCI bleeding-risk model. **eFigure 5** plots the false-discovery rates and positive predictive values for each model at the respective thresholds, showing an improvement in both scenarios.

XI. Limitations in Implementation

Implementing these models for clinical use has been shown to be practical in a number of settings. For example, Huang et al. discussed implementation details of their prediction of acute kidney injury, citing that such implementations were possible if the appropriate fields were extracted from the electronic health record, and gave an example of a real-time risk calculator being implemented in the Cleveland Clinic.[9] However, extraction of such variables may be a limitation and require advanced techniques such as natural language processing to properly extract the needed variables. Even with this limitation, the curated data and model presented in this work can still be used for retrospective benchmarking of quality of care, and enhance understanding of when to employ bleeding avoidance strategies in case reviews.

XII. Additional Discussion and Future Directions

A machine learning model's strong discriminatory abilities, present even with incremental improvements using only the top-10 predictors, allow for confident selection of a subset of clinically useful predictive variables. If the collection of extraneous and collinear variables is not desirable, the blended top-10 variable model with a c-statistic of 0.81 performs well. Selecting a small, parsimonious set of predictors selected by the modeling technique could facilitate development of bedside tools that gather pertinent variables from the electronic medical record, calculate relevant scores, and characterize patients' risk profiles in a variety of ways that clinicians could use to better care for patients.

A third enhancement of this work (in addition to the two presented in the main text) is the prospective prediction as demonstrated in the top-predictors method. By selecting risk thresholds and evaluating treatment vs. non-treatment cases, it is possible to compare risk models with how they would be used clinically. For example, comparing the false discovery rates and quantities of predicted bleeds of the blended model and the blended model using only 10 variables versus the existing NCDR bleeding risk model shows specific improvements with each added layer of complexity. This improvement occurred because gradient descent boosting extracted the full continuous ranges of variables that had previously been used only as dichotomous variables.

© 2019 Mortazavi BJ et al. *JAMA Network Open*.

Dichotomous versions of continuous variables were rarely selected by the model, illustrating the power of gradient descent boosting in selecting its own decision cut-points for continuous variables.

A fourth enhancement is the evaluation of the predictive method in a prospective manner. While the thresholds should vary based upon the use case and costs in each setting the models would be used in, the choice of the data-driven thresholds selected here can greatly reduce the false-discovery rate, which helps reduce treatment by bleeding avoidance therapies by focusing on those at greatest risk, and also reduces the costs associated with mistreatment. The f-score approach is an enhancement beyond the c-statistic discrimination and calibration plots; specifically, if the model is used prospectively, it better pinpoints when to expect a bleeding event and its consequences.

These enhancements allow for the opportunity to extend this work in several areas. The first is to explore enhancements to the bleeding model by considering the further array of available data in the CathPCI registry. Other laboratory values, prior history variables, and values that were not found to be statistically significant in the prior work[1] should be re-evaluated with these machine learning modeling techniques. The second is to explore the dynamic nature of the bleeding risk throughout the patient encounter. Two models were developed in this work, one as a pre-PCI model and one as a post-PCI model before treatment with bleeding avoidance therapies. The data within the CathPCI registry can be split into a variety of key decision points, including choice of access site, choice of bleeding avoidance therapies, and even choice of closure method for femoral PCIs, allowing for multiple models that will show the varying risks before and immediately following key treatment decisions. The third is to extend beyond the bleeding model, applying the techniques presented here to a variety of the models available in NCDR across the registries collected by the American College of Cardiology, evaluating discrimination improvements, identifying predictive factors, and evaluating risk threshold and prospective prediction performance measures.

It will be essential to use electronic medical records to implement machine learning methods if we wish to verify their successes and potential shortcomings in future prospective studies. Registry data are highly curated, and it is unlikely that all potentially pertinent variables for an entire span of a patient's admission would be available for immediate use from the electronic medical record. Two considerations are essential: first, it is important to recognize that electronic medical records have only so many variables readily available, so models built will need to be adjusted to maximize the variables. Second, it may be that certain variables in the registry matter greatly and are not available within the electronic medical record, and should be identified specifically.

References

1. Rao SV, McCoy LA, Spertus JA, et al. An updated bleeding model to predict the risk of post-procedure bleeding among patients undergoing percutaneous coronary intervention: a report using an expanded bleeding definition from the National Cardiovascular Data Registry CathPCI Registry. *JACC: Cardiovascular Interventions.* 2013;6(9):897-904.
2. Montalescot G, Salette G, Steg G, et al. Development and validation of a bleeding risk model for patients undergoing elective percutaneous coronary intervention. *Int J Cardiol.* 2011;150(1):79-83.
3. Chhatriwalla AK, Amin AP, Kennedy KF, et al. Association between bleeding events and in-hospital mortality after percutaneous coronary intervention. *Jama.* 2013;309(10):1022-1029.
4. Chen T, Guestrin C. Xgboost: A scalable tree boosting system. Paper presented at: Proceedings of the 22Nd ACM SIGKDD International Conference on Knowledge Discovery and Data Mining2016.
5. Friedman J, Hastie T, Tibshirani R. Regularization paths for generalized linear models via coordinate descent. *Journal of statistical software.* 2010;33(1):1.
6. Robin X, Turck N, Hainard A, et al. pROC: an open-source package for R and S+ to analyze and compare ROC curves. *BMC bioinformatics.* 2011;12(1):1.
7. Wood SN. *Generalized additive models: an introduction with R.* Chapman and Hall/CRC; 2017.
8. Zeileis A. Econometric computing with HC and HAC covariance matrix estimators. 2004.
9. Huang C, Murugiah K, Mahajan S, et al. Enhancing the prediction of acute kidney injury risk after percutaneous coronary intervention using machine learning techniques: A retrospective cohort study. *PLoS medicine.* 2018;15(11):e1002703.

eTable 1. Model Results for Patient Cohorts[a]

Timing	Variable Set	Logistic Regression	Xgboost	Best f-score
Pre-PCI	Existing NCDR	0.77 (0.77-0.77)	0.81 (0.81-0.82)	0.30 (0.30-0.30)
Post-PCI	Existing NCDR	0.78 (0.78-0.78)	0.78 (0.78-0.78)	0.26 (0.26-0.26)
	Blended NCDR	0.78 (0.78-0.78)	0.82 (0.82-0.82)	0.31 (0.31-0.31)

[a]Best f-score comes from best model (logistic regression or xgboost by mean c-statistic).
NCDR = National Cardiovascular Data Registry

eTable 2. Blended Xgboost Model Incremental Variable Importance

Rank	Feature	Forward, Stepwise C-statistic
1	Pre-procedure hemoglobin	0.635
2	PCI status and cardiac shock composite	0.754
3	Coronary artery disease presentation	0.765
4	Glomerular filtration rate	0.792
5	Female	0.801
6	Body mass index	0.804
7	Age	0.806
8	Cardiogenic shock within last 24 hours or at start of PCI	0.806
9	Cardiac arrest within 24 hours	0.808
10	New York Heart Association composite	0.809
11	Cardiogenic shock within last 24 hours	0.809
12	SCAI lesion class composite	0.812
13	Pre-PCI left ventricular ejection fraction %	0.813
14	PCI status composite	0.813
15	Pre-procedure creatinine	0.813
16	PCI lesion composite	0.814
17	Vessel disease composite	0.815
18	Previous PCI	0.815
19	Stenosis prior to treatment	0.815
20	Pre-procedure TIMI flow	0.815
21	Chronic lung disease	0.816
22	Pre-procedure TIMI, no	0.816
23	Peripheral vascular disease	0.816
24	Cerebrovascular disease	0.817
25	Diabetes: treatment composite	0.817
26	Lesion complexity	0.817
27	Diabetes	0.817
28	PCI status and shock composite 5 - Urgent	0.817
29	PCI status and shock composite 4 - Emergent	0.817
30	Cardiogenic shock at start of PCI	0.817
31	Currently on dialysis	0.817
32	Lytics before PCI for STEMI	0.817
33	Subacute stent thrombosis	0.817
34	Cardiogenic shock composite	0.817
35	Diabetes with non-insulin treatment	0.817

© 2019 Mortazavi BJ et al. *JAMA Network Open*.

36	Left main PCI	0.817
37	NYHA Class is I, II, or III	0.817
**Rank**	**Feature**	**Forward, Stepwise C-statistic**
38	PCI status and shock composite 3 – Shock within 24 hours or at start of PCI	0.817
39	Mild chronic kidney disease	0.817
40	Moderate chronic kidney disease	0.817
41	PCI status and shock composite 2 – Salvage or shock	0.817
42	Chronic kidney disease composite	0.817

Not selected: STEMI, age ≤70, age >70, BMI ≤30, insulin requiring diabetes, no chronic kidney disease 1, severe chronic kidney disease, PCI and shock composite 1, PCI and shock composite 6, SCAI lesion class II or III, SCAI lesion class IV, proximal LAD PCI, NYHA is class IV, 2- or 3-vessel disease, pre-procedure hemoglobin ≤13 g/dL, pre-procedure hemoglobin >13, cardiogenic shock within 24 hours and at start of PCI

CAD, coronary artery disease; GFR, glomerular filtration rate; LAD, left anterior descending; NYHA, New York Heart Association; PCI, percutaneous coronary intervention; SCAI, Society for Cardiovascular Angiography and Interventions; STEMI, ST-segment elevation myocardial infarction; TIMI, thrombolysis in myocardial infarction

eTable 3. Blended Model Dataset Features; Average Rank in 5-fold cross-Validation and Times in Top 10

Rank	Feature	Average Rank (SD)	% in Top 10
1.	Pre-procedure hemoglobin	1 (0)	5
2.	PCI status and cardiac shock composite	2 (0)	5
3.	Coronary artery disease presentation	3 (0)	5
4.	Glomerular filtration rate	4 (0)	5
5.	Female	5 (0)	5
6.	Body mass index	6 (0)	5
7.	Age	7 (0)	5
8.	Cardiogenic shock within last 24 hours or at start of PCI	9.4 (2.07)	4
9.	Cardiac arrest within 24 hours	8.4 (0.55)	5
10.	NYHA composite	10.6 (1.14)	2
11.	Cardiogenic shock within the last 24 hours	11.2 (2.17)	3
12.	SCAI lesion class composite	12.2 (0.45)	0
13.	Pre-PCI LVEF %	11.6 (1.34)	1
14.	PCI status composite	14.6 (0.55)	0
15.	Pre-procedure creatinine	14 (0.71)	0
16.	PCI lesion composite	16 (0)	0
17.	Vessel disease composite	17 (0)	0
18.	Previous PCI	19.2 (0.84)	0
19.	Stenosis prior to treatment	18.4 (0.55)	0
20.	Pre-procedure TIMI flow	19.6 (1.14)	0
21.	Chronic lung disease	20.8 (0.45)	0
22.	Pre-procedure TIMI, no	23.6 (1.82)	0
23.	Peripheral vascular disease	22.6 (0.89)	0
24.	Cerebrovascular disease	23.4 (0.55)	0
25.	Diabetes: treatment composite	24.8 (0.84)	0
26.	Lesion complexity	25.6 (0.55)	0
27.	Diabetes	27 (0)	0
28.	PCI status and shock composite 5 - Urgent	28.2 (0.45)	0
29.	PCI status and shock composite 4 - Emergent	29.2 (0.84)	0
30.	Cardiogenic shock at start of PCI	30.4 (1.67)	0
31.	Currently on dialysis	32.2 (1.30)	0
32.	Lytics before PCI for STEMI	33.6 (0.55)	0
33.	Subacute stent thrombosis	32.6 (0.89)	0
34.	Cardiogenic shock composite	30.8 (0.84)	0
35.	Diabetes with non-insulin treatment	35.4 (0.55)	0
36.	Left main PCI	35.6 (0.55)	0
37.	NYHA class is I, II, or III	37 (0)	0
38.	PCI status and shock composite 4 - Shock	38.2 (0.45)	0
39.	Mild chronic kidney disease	40.4 (0.89)	0

40.	Moderate chronic kidney disease	39 (0.71)	0
41.	PCI status and shock composite 2 – Salvage or shock	40.4 (0.55)	0
42.	Chronic kidney disease composite	42 (0)	0

LVEF, left ventricular ejection fraction; NYHA, New York Heart Association; PCI, percutaneous coronary intervention; SCAI, Society for Cardiovascular Angiography and Interventions; SD, standard deviation; STEMI, ST-segment elevation myocardial infarction; TIMI, thrombolysis in myocardial infarction

eTable 4. Blended Model Dataset Features; Stepwise Forward Selection C-statistic in 90/10 Training/testing Split.

Rank	Feature	Forward, Stepwise C-statistic
1.	Pre-procedure hemoglobin	0.634
2.	PCI status and cardiac shock composite	0.753
3.	Coronary artery disease presentation	0.763
4.	Glomerular filtration rate	0.790
5.	Female	0.800
6.	Body mass index	0.804
7.	Age	0.805
8.	Cardiogenic shock within last 24 hours or at start of PCI	0.805
9.	Cardiac arrest within 24 hours	0.807
10.	NYHA composite	0.808
11.	Cardiogenic shock within the last 24 hours	0.808
12.	SCAI lesion class composite	0.811
13.	Pre-PCI LVEF %	0.812
14.	PCI status composite	0.812
15.	Pre-procedure creatinine	0.812
16.	PCI lesion composite	0.813
17.	Vessel disease composite	0.814
18.	Previous PCI	0.814
19.	Stenosis prior to treatment	0.814
20.	Pre-procedure TIMI flow	0.815
21.	Chronic lung disease	0.815
22.	Pre-procedure TIMI, no	0.815
23.	Peripheral vascular disease	0.815
24.	Cerebrovascular disease	0.816
25.	Diabetes: treatment composite	0.816
26.	Lesion complexity	0.816
27.	Diabetes	0.816
28.	PCI status and shock composite 5 - Urgent	0.816
29.	PCI status and shock composite 4 - Emergent	0.816
30.	Cardiogenic shock at start of PCI	0.816
31.	Currently on dialysis	0.816
32.	Lytics before PCI for STEMI	0.816
33.	Subacute stent thrombosis	0.816
34.	Cardiogenic shock composite	0.817
35.	Diabetes with non-insulin treatment	0.817
36.	Left main PCI	0.817
37.	NYHA class is I, II, or III	0.817
38.	PCI status and shock composite 4 - Shock	0.816
39.	Mild chronic kidney disease	0.816
40.	Moderate chronic kidney disease	0.816

41.	PCI status and shock composite 2 – Salvage or shock	0.816
42.	Chronic kidney disease composite	0.816

LVEF, left ventricular ejection fraction; NYHA, New York Heart Association; PCI, percutaneous coronary intervention; SCAI, Society for Cardiovascular Angiography and Interventions; SD, standard deviation; STEMI, ST-segment elevation myocardial infarction; TIMI, thrombolysis in myocardial infarction

© 2019 Mortazavi BJ et al. *JAMA Network Open*.
